# Hospital and Institutional News

**Published:** 1912-02-24

**Authors:** 


### HOSPITAL AND INSTITUTIONAL NEWS.

### THE CHARITY COMMISSIONERS AND SPALDING HOSPITAL.

A public inquiry into the affairs and management of the Johnson Hospital, Spalding, and of the Spalding Dispensary will be held by the Charity Commissioners in the Council Room at the Corn Exchange, Spalding, next Wednesday, 28th instant, at 5 P.M.

### THE GOVERNMENT'S COMMITTEE ON TUBERCULOSIS.

The Chancellor of the Exchequer is appointing a Committee to draw up a report for the guidance of the Government and local bodies in making provision for the treatment of phthisis in sanatoria or other institutions. The members of this Committee are: Mr. Waldorf Astor, M.P. (chairman); Dr. Christopher Addison, M.P., M.D.; Dr. N. D. Bardswell, M.D., Medical Superintendent of the King Edward VII. Sanatorium, Midhurst; Mr. David Davies, M.P., President of the King Edward VII. Welsh National Memorial Association; Dr. A. Mearns Fraser, M.D., Medical Officer of Health, Portsmouth; Dr. A. Latham, M.D., Physician at Mount Vernon Hospital for Consumption; Dr. W. Leslie Mackenzie, M.D., Medical Member of the Local Government Board for Scotland; Dr. J. C. McVail, M.D., Medical Member of the National Health Insurance Commission (Scotland); Dr. W. J. Maguire, M.D., Medical Member of the National Health Insurance Commission (Ireland); Sir George Newman, M.D., Chief Medical Officer of the Board of Education; Dr. Arthur Newsholme, C.B., M.D., Medical Officer of the Local Government Board; Dr. James Niven, LL.D., M.P., Medical Officer of Health, Manchester; Dr. Marcus Paterson, M.B., Medical Superintendent of the Brompton Hospital Sanatorium, Frimley; Dr. R. W. Philip, M.D., Honorary Examining Physician Queen Alexandra Sanatorium, Davos, and Physician of the Royal Victoria Consumption Hospital, Edinburgh; Dr. H. Meredith Richards, M.D., Medical Member of the National Health Insurance Commission (Wales); Mr. T. J. Stafford, C.B., F.R.C.S.I., Medical Member of the Local Government Board for Ireland; Miss Jane Walker, M.D., Medical Superintendent of the East Anglian Sanatorium, Nayland, Colchester; Mr. J. Smith Whitaker, Medical Member of the National Health Insurance Commission (England). The secretary to the Committee will be Mr. F. J. Willis, one of the Assistant Secretaries of the Local Government Board.

### THE PERSONNEL OF THE COMMITTEE.

The composition of this Committee on Tuberculosis is very obviously a compromise. Political and party considerations clearly have led to the inclusion of certain names which it is unnecessary to repeat. On the other hand, as Mr. R. Clement Lucas, F.R.C.S., has promptly pointed out, a Committee on Tuberculosis which includes the name of no surgical authority, and which apparently is to confine its attention to "one form of a very general infecting disease," can hardly be called a committee of experts. We can only hope that before the Committee has an opportunity of affecting the course of procedure these points will be considered seriously, or reason be shown why Mr. George has chosen to ignore them at the outset.

### STAFF CHANGES AT LONDON MENTAL HOSPITALS.

There have recently been several changes in the staffs of the various London mental hospitals. Dr. Hubert Bond has resigned his appointment as Medical Superintendent of Long Grove Mental Hospital on his appointment by the Lord Chancellor to be a Commissioner in Lunacy. The Mental Hospitals Committee of the London County Council, reporting on his resignation, states that Dr. Bond has been in their service for 19 years, and has been Medical Superintendent of the Epileptic Colony as well as of Long Grove Mental Hospital. At both these institutions his care and forethought for the patients, for the welfare of the staff, and his thorough administration have earned the Committee's high approbation, and it offers its congratulations to Dr. Bond on his new appointment. Mr. R. H. H. Jolly having resigned his post as Fifth Assistant Medical Officer at Long Grove Mental Hosptal, Mr. H. W. White has been transferred from the position of Junior Assistant at the Manor Mental Hospital to fill the vacancy. There is, by the way, still a vacancy for a Sixth Assistant at Long Grove. At Horton Mental Hospital, Mr. Laurence Gavin has resigned the post of Third Assistant Medical Officer on his appointment as Medical Superintendent of the Mullingar District Mental Hospital. The Fourth, Fifth, and Sixth Assistants have been promoted each a grade, and Mr. P. S. Vickerman has been appointed to fill the vacancy for Sixth Assistant.

### LORD LISTER AS HOSPITAL PRESIDENT.

The supporters of St. Mary's Hospital for Women and Children, Plaistow, E., were reminded at the annual court of the great loss which this institution in particular sustained in the death of the late Lord Lister, who for many years was the president of the hospital. Towards the end of April last, it should be recalled, the in-patients were transferred from the old building to the new ward block, greatly to their benefit. The wards in the old building have been remodelled and now provide accommodation for the entire nursing staff, and the old system of boarding them away from the hospital has been given up. The Viscountess Parker, an energetic supporter of the hospital, very kindly defrayed the cost of these alterations. The committee also referred to the generous support which has been accorded to the institution by the large London funds. The Million Pennies Fund, which is being raised by the matron, Miss Kate L. Ray, for the provision of a nurses' home and out-patient department, we understand, is making good progress. The poor accommodation for the nurses and the overcrowded state of the waiting-room for the patients require to be brought up to date as soon as funds permit. An $x$-ray apparatus has been given by Mr. and Mrs. H. O. Holford, and will be installed shortly. The garden-roof, which, by the way, is a feature of the new hospital, has during the past summer been greatly appreciated by the patients, and especially by the children. Accommodation for twenty patients is still waiting for funds to enable it to be occupied.

### KIDDERMINSTER'S NEW MEDICAL OFFICER.

Mr. W. Hodgson Moore has been elected Medical Officer of Health for Kidderminster, subject to the approval of the Local Government Board. Mr. Moore, a Guy's man, took his M.R.C.S. in 1883 and his L.R.C.P. in 1896. His previous appointments include those of Medical Officer to the Kidderminster Workhouse, and Hon. Surgeon to the Kidderminster Infirmary and Children's Hospital.

### A HOSPITAL SECRETARY AND THE INSURANCE ACT.

A possibly memorable rule was passed last week at the annual meeting of the Sussex Eye Hospital, on the proposal of Colonel Somers Clark, the honorary secretary. It was in these terms: "No person who is entitled to free medical or surgical assistance under any Act of Parliament, Order in Council, or Order of a Government Department or local authority, or from the State or any organization, body, authority, society, company, association, or person wholly or partially financed by the State or out of the rates, is entitled to the benefits of this Institution, but the Committee of Management may, if they think fit, from time to time make, and afterwards vary, or rescind, rules for the admission of all or any such persons or classes of persons." This rule, apparently, is, in idea, a sequel to the resolution Colonel Somers Clark believed had been passed by every medical charity subject to the Sussex Hospital Board, not to treat except in special cases children sent by the school medical officer. The Colonel explained the rule as defensive merely, and was followed by Mr. H. H. Taylor, who instanced compulsory education and compulsory vaccination as proofs that when the State insisted on anything being done it should be responsible for paying for it. Such action as the passing of this rule shows that under the apparent quiet which appears to pervade the institutional as opposed to the medical world, a strong feeling is being aroused, and it remains to be seen whether, should the call for an amended Act be disregarded, such defensive actions generally will be decided upon.

### A BATH DISPENSARY AND THE INSURANCE ACT.

Individual initiative deserves to be recorded wherever it appears, so we think it worthy of note that Mr. P. H. Ryan, hon. medical officer to the Southern Dispensary, Bath, has made his report of public importance by including in it a table showing how the Insurance Act will affect the district which his dispensary serves. "It will be seen," Mr. Ryan says by way of introduction, "that the number of men between 16 and 70 who will be definitely included in the Act is just over 10 per cent. Of women over 16, together with men over 70 (50 per cent.) the larger number will be excluded, and the children up to 16 (40 per cent.) will be all definitely excluded. At a rough estimate, then, the dispensary will still have to cater for 75 to 80 per cent. of its present numbers." This is enough to show the value of the work which a dispensary performs, and Mr. C. S. Elwell, the hon. secretary, should be able by it to enforce a very strong local claim to public support.

### A DOCTOR'S COTTAGE HOSPITAL.

The late Dr. J. Kershaw's recent bequest of £40,000 for the foundation of a hospital at Royton, near Oldham, on the chances of fulfilling which we commented at the end of December, is apparently to be devoted to a cottage hospital. This means that the onus of raising an income from voluntary sources will not fall upon the Hospital's committee, as the sum left over for endowment purposes will be more or less sufficient for its maintenance. A committee is now conferring with the architect, and Dr. Young, the medical officer of health, has been asked, we understand, to reinforce the committee with some of the local practitioners.

### WORKING-MEN AND PONTYPOOL HOSPITAL.

Anybody who reads a report of the annual meeting last week of the Pontypool and District Hospital will readily understand the jubilance of the secretary, Mr. T. Morgan, whose letter on the value of working men's support we publish this week. The ordinary income for the past year amounted to £3,078, or to £378 more than its predecessor, an improvement gratifying enough to a secretary who, like Mr. Morgan, has, we believe, been appointed within the last twelve months. The working-men's contributions, moreover, have risen to £2,000, and to them is due the chief praise for the hospital's balance in hand. Mr. J. C. Hanbury, D.L., is again president, and Mr. Evan Price, Llanerch Colliery, and Mr. Tom Green, Blaendare Colliery, have filled the two vacancies on the Colliery Employees' section. Mr. T. Langley raised the question whether it was fair that all the governors attending the annual meeting should take part in voting on the miners' candidates. It was not clear from the report exactly what grievance Mr. Langley had in mind. Mr. G. Jenkins, J.P., who was in the chair, did his best to reassure Mr. Langley, but we should be glad to have further information elucidating the rights and wrongs of his point of view.

### SHEFFIELD CHAPLAINS AND "THE HOSPITAL."

According to the *Sheffield Daily Telegraph*, the Sheffield clergy are indignant at our suggestion that the decadence of Hospital Sunday can be accounted for only by lack of enthusiasm on their part. The Rev. W. J. Cole is quoted as criticising our statement in the phrase "a gross libel," but he did not apparently discuss the matter further, and yet were the clergy, Mr. Cole included, able to explain to what else the falling-off in Hospital Sunday subscriptions is due, we should be very glad to publish so important an explanation. For clearly an explanation of a failure is the first step towards a remedy. We are therefore glad to quote the explanation attributed to the Rev. J. G. Williams, who is reported to have said: "The clergy act, but the people don't give. Many old families that gave nobly have died out. New people come to the parish, but give nothing. Others . . . plead . . . Lloyd George finance . . . "

### THE REV. J. G. WILLIAMS' EXPLANATION.

This is instructive as far as it goes, but, as we believe Mr. Williams will admit on reflection, his statements are excuses and not arguments. They are excuses, not in the least because we imagine them to be offered in a self-protecting spirit, but because they are the old ones habitually pleaded by every hard-up charitable movement in the country, in spite of the fact that individual charities are as prosperous as ever. They are not arguments, still less explanations, because if they were, the whole voluntary system must have broken down before this. Yet the startling fact remains that while the whole system of voluntary support, if not indeed stronger, is at least as strong as it was ten years ago, Hospital Sunday, which used to be so magnificent a pillar of the voluntary system, is to-day bending like a broken reed. We invite Mr. Williams to probe the subject a little deeper, and as our presentation of his case this week will show, are genuinely anxious to have the matter threshed out fully.

### THE BOSTON HOSPITAL'S GLOOMY OUTLOOK.

The prospects of carrying out certain important reforms, such as a new mortuary, for instance, at Boston Hospital, Lincolnshire, do not appear to grow greater as time goes on. A recent specially called meeting revealed to the apparently astonished chairman, Mr. W. Porter, a bundle of bills amounting to £50, and, on inquiry from Mr. Cooke-Yarborough, the honorary secretary, an overdraft at the bank of £216. "If," said Mr. Porter dismally, "I had anticipated we should be in anything like the position we are in when I spoke in another place on behalf of a series of alterations involving an outlay of £500, I should not have advocated it"; and again, "the managers are not prepared to carry out several alterations suggested by Sir Henry Burdett because of the want of funds."

### A PRACTICAL SUGGESTION FOR RAISING MONEY.

On December 16 last we reported the formation of a sub-committee to consider the alterations that were virtually accepted as necessary after our Report had appeared in the first week of November. To-day another sub-committee has been formed, consisting of Mr. Porter, Mr. A. Cooke-Yarborough, and Dr. South, to consider with the matron the desirability of reducing the number of beds to six in the men's and women's and to three in the children's wards. Mr. Z. Cheshire, Mr. Cooke-Yarborough, and Canon Heygate infused some life into these counsels by stating, what all experience shows to be the truth, that the success of a hospital appeal depends chiefly on the amount of brains and energy put into its organisation. Mr. Cheshire suggested a bazaar; the honorary secretary made the still more practical suggestion that a Ladies' Hospital Association should be formed. There is no doubt he is right. This should be undertaken at once, and, as he pointed out, if Mrs. Gilliatt can collect more than £10 in one district, 20 or 30 ladies of equal energy can do the same. By this suggestion the meeting was saved a little from the gloom with which it opened. Such a suggestion as the Secretary's will do more, if followed up, than all the reductions of beds and the raising of fees ever put forward. Will it be pressed forward? If not, no one could blame Mr. Cooke-Yarborough for resigning in disgust. We hope instead of this that Mr. Cheshire and Canon Heygate will spare no trouble to assist him in insisting that a Ladies' Association shall be formed at once.

### THE NEW MAUDSLEY MENTAL HOSPITAL.

In answer to an inquiry from the Lunacy Commission as to the name by which it is proposed that the mental hospital to be built at Denmark Hill shall be known, the Asylums Committee of the London County Council communicated with Dr. Henry Maudsley. In his reply, Dr. Maudsley stated, to the Committee's great pleasure, that he does not object to the use of his name in the title for the new institution. The Committee has therefore determined that the hospital shall be known as "The Maudsley Hospital." The Committee is very glad thus permanently to associate Dr. Maudsley's name with the hospital which owes its establishment to his timely suggestion and towards the cost of which he has contributed very generously.

### SOME SATURDAY FUND APPOINTMENTS.

In view of the successful dinner which the Saturday Fund held last week its supporters will be interested in the officers appointed by the Board of Delegates. Sir Savile Crossley is again chairman of the Fund, with Mr. H. Gower as chairman of the Council. The Hon. H. L. W. Lawson, M.P., has been appointed honorary treasurer, and Mr. B. Marwood honorary sub-treasurer. By the way, Hospital Saturday for this year has been fixed for October 12.

### A SUCCESS FOR THE WORTHING HOSPITAL.

The opening of the new out-patient department at this institution last week was a real triumph for the medical staff, the committee, and the secretary, Captain H. J. Prentice. The two latter were fortunate in securing the Duke of Norfolk to perform the opening ceremony, and to complete the ceremonial character of the occasion by the novel addition of a play, the author of which, Mrs. Ayton Gosling, is the wife of the consulting surgeon to the hospital, Mr. Ayton Gosling. Mr. W. S. Simpson, the senior medical officer, made a capital speech, which emphasised the great difficulties of the medical staff in dealing with cases in the old out-patient department.

### THE ARCHITECT AND HIS DESIGN.

Mr. Simpson also boldly appealed for an *x*-ray and an electrical apparatus, and gave some well-deserved thanks to the Mayor, Alderman E. C. Patching, J.P., for his initiative. The building itself has been designed by Mr. A. Morris Butler, A.R.I.B.A., and its ground floor consists of a hall, a dispensary, two consulting-rooms, a special room for eye cases, and the secretary's office. On the first floor are nurses' bedrooms, bathrooms, and a linen store. We understand that "Kentobin" flooring has been used, while the walls of the hall are of white glazed tiles. The chairman of the hospital committee, Mr. H. R. P. Wyatt, thanked the Duke for coming, and we hope that the second half of the £3,000 required will soon be subscribed.

### THE LORD MAYOR AS AN OPERATOR.

The Lord Mayor, Sir Thomas Crosby, M.D., who by the way has just had another honour, the Honorary LL.D. of St. Andrews, conferred upon him, lent a peculiar interest to the annual meeting of St. Mark's Hospital for Fistula last week by recalling his own practical experience in the theatre of that institution. Sir Thomas Crosby said that he had known and valued the hospital's work for fifty years, and that he had assisted on many occasions at operations in the hospital. The secretary, Mr. A. W. Sowden, must be grateful for Sir Homewood Crawford's appeal for an increased grant from the King's Fund, and is to be congratulated that while the constitution of the hospital lays down that the president shall be the Lord Mayor, this year that office fortunately is held by a medical man. It must be gratifying to the Lord Mayor also to return as Chief Magistrate of the City to support the claims of the hospital where he worked in the past.

### APPOINTMENTS AT WEST HAM HOSPITAL.

Dr. McIntyre has been appointed Senior House Surgeon; Dr. D. S. Craig, Junior House Surgeon, and Mr. Smith, Assistant Dispenser at West Ham. Mr. A. Govier, J.P., who is not offering himself for re-election to the committee, has been appointed a Vice-president. Mr. Govier's experience of hospital work at West Ham has been not only a long one, for he has seen it grow from its early years; not only a worker's one, for he has been active on the committee for much of this period; but also the most intimate possible, since he himself has been a patient in its wards. The monthly meeting at which these appointments were confirmed was of more than ordinary interest by the reminder of the chairman, Mr. T. A. Cook, that Lord Lister was born in West Ham, and by explanations offered by the medical staff of certain statements that have been made recently, and it seems given a certain currency by the coroner, Dr. Ambrose. A letter from a grateful patient, Mrs. Mearns, was read also, the writer showing her gratitude by undertaking to form a working party for the hospital at Brentwood.

### DR. JEX-BLAKE'S "LIFE" TO BE WRITTEN.

All interested in the pioneer work of the late Dr. Sophia Louisa Jex-Blake, M.D. (Berne), a record of whose career recently appeared in our columns, will learn with pleasure that her life is to be written. Her biographer is to be Dr. Margaret Todd, M.D. (Brux.), formerly Dr. Jex-Blake's assistant medical officer at the Edinburgh Hospital and Dispensary for Women and Children. Dr. Todd will be grateful "if old friends will send letters or particulars of Dr. Jex-Blake's early years," and undertakes to return all communications, if so desired, after making a copy of them. All such information should be addressed to Dr. Todd at Windydene, Mark Cross, Sussex. In this connection it is also interesting to add that Dr. Jex-Blake's will is now published, and that she left estate valued at £14,196 gross and £11,095 net. Her bequests, we may note, included £100 to the Society for Promoting the Employment of Women, and £100 to one of the Women's Suffrage Societies.

### LENDING AN OPERATING-TABLE.

A new operating-table was recently purchased for the Lewisham Poor Law Infirmary. With regard to the old one at first it was suggested, we are informed, that it should be sent to the carpenter's shop. Mr. Alfred O. Weeks, however, opposed this, as he held there were plenty of infirmaries that might be in need of one, but which were not so particular as Lewisham in having the very latest pattern. Since then, we understand, Mr. Weeks induced the Guardians to offer the old operating-table for acceptance to St. John's Hospital, Morden Hill, the Home for Sick Children, Sydenham, or to the Eltham Cottage Hospital. No doubt there are numerous small hospitals and infirmaries, dependent on public subscriptions and not on the rates for their support, where similar gifts would be acceptable, and consequently the example of the Lewisham Guardians might well be followed.

### ONE WAY OF GETTING ANNUAL SUBSCRIPTIONS.

An ingenious, if reprehensible, mode of gaining annual subscriptions, is given in Mr. Godfrey H. Hamilton's excellent and amusing paper on "Hospital Appeals," which is published in the current *Hospital Gazette*. Quoting a confession made to him by a retiring secretary whose institution had been noticeably to the fore with annual subscriptions for some years, Mr. Hamilton gives his secret: "Appeal without ceasing, call all amounts received of £5 5s. and under Annual Subscriptions, apply for them the second year, and you will be surprised to find what a number pay up. Many are annoyed, but it is easy to apologise for a slip, and though a number do not repeat their gifts you are constantly filling their places by new donors." Mr. Hamilton naturally only recommends the asking of all subscribers to repeat their gifts about the anniversary of their previous donation.

### A NEW LIGHT ON INSURANCE AND CHARITY.

We cannot forbear to notice two other questions on which Mr. Godfrey Hamilton has some interesting things to say. The first is the proposal for some combination of insurance and charity. To quote once more: "It sometimes happens that a person interested in a hospital wishes to do something, but cannot afford substantial help out of income. A hospital, by taking over part of his capital, can well afford to pay him, after investing in trustee securities, three to six per cent., according to age, for the rest of his life and to come out a considerable gainer. At the death of such a donor, however, Somerset House receives legacy and succession duties amounting together to about 1.5 per cent. of the capital sum. This system of donations carrying life annuities was, I believe, instituted by Mr. Burford Rawlings." The whole paper, however, is worth careful reading, but we must remember that this subject of insurance and charity is one that necessarily finds its most likely support in hospitals which deal exclusively with paralytic and epileptic patients.

### A HOSPITAL CHAPLAIN'S OPPORTUNITIES.

We see from the annual report that the Rev. E. H. Nash, M.A., Vicar of St. Paul's Church, is the successor, as chaplain to the Chichester Infirmary, of the Rev. Edward Bray, who has held the post of chaplain for twenty-eight years. In these days of the decline of Hospital Sunday, as we fear the reduced subscriptions to this movement all over the country must be called, the office of chaplain is taking on in one sense a new responsibility. The office of chaplain generally goes hand in hand with a particular incumbency, and a movement is very rightly taking place whereby the duties of the hospital chaplain, in attracting support outside to the hospital, are being held as no less part of the office than the visiting of the sick inside the wards. There is no doubt, therefore, that recently appointed chaplains have a great opportunity as men who come with a fresh inspiration to the work of resuscitating Hospital Sunday, not merely in the affections of their congregations, but on all occasions that may occur. A hospital chaplain in a profound sense is a hospital missionary. He adds to his duties as rector a personal knowledge of hospital work and the status of a hospital official. Thus he should remember, almost as invariably as the Hospital Secretary is trained to do, his institutional duty to his hospital whenever he ascends the platform. One side of the success of the chaplain's work is now being tested by the success of local Hospital Sunday movements. Few if any officials have a finer opportunity than hospital chaplains to-day of doing a needed and often neglected work.

### SERVANTS' HOMES FOR DOMESTIC STAFFS.

The second number of that new Canadian periodical, the *Hospital World*, contains, besides a reprint of the proceedings of the Conference of the British Hospitals Association at Manchester last September, a plea for the establishment of servants' homes. "The time has come," says this journal, "for the establishment of servants' homes just as we now have nurses' homes, where the toilers, male and female, in hospital wards may get away from the atmosphere of the sick for twelve hours, have comfortable single bedrooms, common rooms for reading and recreation, plenty of bathrooms, and ordinary home comforts." Hospital secretaries in England will probably feel at first a little aghast at the heavy outlay such a scheme would cost, and of the increased complexity of administration which it might necessitate. Nevertheless the negative side of the proposal is unfortunately more or less true, that, namely, the accommodation of the domestic staff is often not in keeping with the hygienic requirements of comfort and privacy desirable in every department of a modern hospital to-day.

### A HOSPITAL OF MANY SITES.

In order to appreciate the importance of the recent opening by Lord Derby of the High Street branch of St. Mary's Hospital, Manchester, a glance backward is necessary. St. Mary's Hospital was established as the Manchester and Salford Lying-in Hospital on Salford Bridge in 1790, practically 122 years ago. From 1796 to 1840 it was at Stanley Street, New Bailey Street, whence it was removed to South Parade, Manchester. From 1856 to 1904 the hospital was in Quay Street, Manchester, since when its work has been carried on at the Whitworth Street Hospital, near Oxford Road Station, the foundation-stone of which was laid by the Countess of Derby on October 9, 1899, and the building was opened for patients in June 1904. Since 1856 the treatment of special diseases has occupied a more and more prominent position. This hospital, we believe, is the oldest hospital in Manchester for sick children—it has the important claim to be the first where lectures and systematic instruction to nurses, midwives, and pupils were given; indeed, courses of lectures on midwifery to midwives and female pupils have been continuously delivered from its foundation to the present time. It is believed that it was one of the first lying-in hospitals to permit medical students to attend the practice of the hospital.

### HOW ST. MARY'S HOSPITALS AMALGAMATED.

The Southern Hospital was established in Grosvenor Street, C.-on-M., in 1886, and later it was removed to Clifford Street, where at first there were twelve beds. Great extensions made no fewer than forty beds there, and the maternity beds, numbering twelve, had been meanwhile (in 1887) transferred to Upper Brook Street as the Manchester Maternity Hospital. In 1908 all these institutions were amalgamated under the title of "The St. Mary's Hospitals" and carried on their work at Whitworth Street, Manchester. The new hospital (known as the High Street Branch) has been built, furnished, and equipped to the memory of the late Manasseh Gledhill by his sole surviving trustee, Mr. Reid. In accordance with Mr. E. Hopkinson's offer to give £2,000 and several other generous gifts conditionally that it should be ready for patients on April 18, 1911, the children were all transferred on that date from the Whitworth Street West Branch. The Whitworth Street Hospital has been completely repainted and decorated and rearranged, including provision for eleven students and thirty-four pupil midwives. For the future the work is apportioned between the two buildings in such a way that at Whitworth Street there are at least forty beds for maternity cases and the out-patients' department, while at the new hospital there will be eighty-four beds for dealing with diseases of women apart from maternity cases, twenty-five beds for children, and two for isolation purposes. Special note should be made of the facilities provided for medical education.

### THE NEW SECRETARY FOR BINGLEY COTTAGE HOSPITAL.

We intended, but for pressure of space last week, to draw the attention of the new secretary of the Bingley Cottage Hospital, Mr. J. H. Sampson, to the proposals of the Acton Cottage Hospital, already alluded to in our columns, for dealing with the Insurance Act. He undertakes the secretaryship at a very important time in hospital history, at a time when the previous secretary, Mr. W. W. Platts, may well be glad to lay down duties for which he has been responsible during twelve years. Mr. Sampson's attitude on the Insurance Act gains in significance from the fact that Bingley Cottage Hospital is a relatively modern one, being only 22 years old, and has already accommodation for 21 patients. In other words, this hospital is likely to grow with its district, and any Insurance Act that may eventually survive its present necessary amendment is bound to affect such institutions as that at Bingley with increasing pressure in a few years' time.

### AN INSPIRING COTTAGE HOSPITAL CHAIRMAN.

When the new children's ward and the new accident ward at the Wallingford Cottage Hospital were recently opened, in spite of the generous emphasis of Mr. P. de M. Cavell, J.P., the hon. secretary, it was hardly realised locally how much the extension had been due to the active enthusiasm of one man. That man was the hospital's chairman, Mr. Wormald. First of all, the attempt to include the children's ward in the town's Coronation scheme was frustrated, and, secondly, an appeal for the out-patient department had been issued not so very long ago. In other words, it was a moment of depression, when energy was slackening and hopes were few. All hospital secretaries appreciate this moment and the paralysis it produces. A sub-committee was formed, but success was still far off and despondency increasing when Mr. Wormald jumped up in his capacity of chairman, and promised to collect the £450 required himself. As often before, one man did what a sub-committee had failed to do, and Mr. Wormald's service was of that invaluable kind which attracts enthusiastic disciples. Miss Hedges, who gave the children's cots and previously £100, Mr. Faber, and Mr. Fraser and Captain Morrison, together with Mr. and Mrs. Potter—these are they who responded promptly and generously to their chairman's energetic example. These facts are worth mentioning, because they consolidate local interest in a hospital, and attract to it a band of workers whose own past efforts create a personal and ultimately a local pride in the institution. It is of such material in fact that the whole voluntary system has been built up.

### IRISH HOSPITALS AND THE INSURANCE ACT.

"Although Ireland has been excluded from medical benefits under the Insurance Act, yet the application of the rest of it to Ireland affects so many people through compulsory contributions that apprehension is felt that Insurance contributors will relax their generosity to hospitals on that account, besides which the Board will have to contribute under the Act being employers." The above interesting extract is taken from the latest annual report of the South Infirmary, Cork, and in connection with it our readers should be reminded of the proposed amendments to the Act which the Conference of Local Hospitals at Cork, reported in The Hospital, passed in the interest of Irish hospitals. Perhaps the chief recommendation was: "That we, as representing the Cork hospitals, express our opinion that the voluntary hospitals be excluded from contributing under the Bill; or, in the alternative, that power be given to the Insurance Commissioners to pay the contributions of employer in addition to the Imperial contributions in the cases of voluntary hospitals out of the funds at their disposal." The foregoing amendment was, of course, not included in the Act.

### THIS WEEK'S DRUG MARKET.

A number of price changes—most of them of minor importance—have taken place since our last report appeared, but business in drugs is still far from brisk. Cod-liver oil continues to be one of the chief articles of interest, and as the reports from Norway are of a favourable character the downward tendency in price is maintained. As was anticipated, a small advance in the price of quicksilver has taken place, and in consequence makers of mercurials have announced a slight increase in their quotations for calomel, corrosive sublimate, and other salts of mercury. Makers of potassium iodide have raised their prices by sevenpence per ounce: the advance, which was somewhat unexpected, is due to an absence of competition; resublimed iodine, iodoform, and other preparations of iodine are unchanged. Drugs the prices of which are tending in an upward direction are menthol, liquorice root, ipecacuanha, jalap, hydrastis, canadensis, cascara sagrada, orange peel, chamomiles, and essence of lemon. Those which are tending downwards in price are sugar of milk, oil of cloves, and cream of tartar. Carbolic acid also shows a slight tendency towards lower prices, and there is now less reason to expect an advance in the quotations for salicylic acid and sodium salicylate. The high value of opium is well maintained and there is no alteration in the prices of morphine and codeine. A further advance in the price of santonin is by no means improbable, notwithstanding the extremely high figure already quoted.

